# OsmiR159 Modulate BPH Resistance Through Regulating G-Protein γ Subunit *GS3* Gene in Rice

**DOI:** 10.1186/s12284-023-00646-z

**Published:** 2023-07-04

**Authors:** Yanjie Shen, Guiqiang Yang, Xuexia Miao, Zhenying Shi

**Affiliations:** 1 Key Laboratory of Insect Developmental and Evolutionary Biology, CAS Center for Excellence in Molecular Plant Sciences, Institute of Plant Physiology and Ecology, Chinese Academy of Sciences, Shanghai, 200032 China; 2 University of Chinese Academy of Sciences, Shanghai, 200032 China; 3 Wuzhou Agricultural Product Quality and Safety Integrated Test Center, Wuzhou, China

**Keywords:** Brown planthopper, BPH resistance, OsmiR159, *OsGAMYBL2* gene, *GS3* gene

## Abstract

Brown planthopper (BPH) is the most destructive insect pest to rice that causes tremendous yield loss each year in rice planting Asia and South-East Asia areas. Compared with traditional chemical-based treatment, utilization of plant endogenous resistance is a more effective and environmental-friendly way for BPH control. Accordingly, quite a few quantitative trait loci (QTLs) for BPH resistance were cloned using forward genetics. However, BPH is apt to change quickly into new biotypes to overcome plant resistance, therefore, new resistance resources and genes are continuously needed. miRNAs are important regulators in both plant development and physiological regulation including immunity, and might be used as effective supplements for BPH resistance QTLs. miR159 is an ancient and conserved miRNA. In this study, we found that each *OsMIR159* gene in rice responded to BPH feeding very obviously, and genetic function assay proved them to negatively regulate BPH resistance, with STTM159 showing resistance to BPH, and over expression of OsmiR159d susceptible to BPH. One target genes of OsmiR159, *OsGAMYBL2*, positively regulated BPH resistance. Further biochemical studies revealed that OsGAMYBL2 could directly bind to the promoter of G-protein γ subunit encoding *GS3* gene and repress its expression. And genetically, *GS3* responded to BPH feeding promptly and negatively regulated BPH resistance, *GS3* over expression plants were susceptible to BPH, while *GS3* knock-out plants were resistant to BPH. Thus, we identified new function of OsmiR159–OsGAMYBL2 in mediating BPH response, and revealed a new OsmiR159–G protein pathway that mediates BPH resistance in rice.

**Supplementary Information:**

The online version contains supplementary material available at 10.1186/s12284-023-00646-z.

## Introduction

Rice is one of the most important food crop in the world, providing food for more than one half of the world population (Sasaki and Burr 2000). In China, rice yield account for about 40% of the total crop yield, and therefore remains to be the most important crop. During the growth process, crops are continuously challenged by the ever-changing environment caused by different types of biotic and abiotic stresses. Sustainable yield would help sustain the food supply needed for the growing world population, especially underlying various adversities such as the shrinking arable land, shortage of irrigation water and changing environments, especially global warming worldwide (Salehi 2022; Xu et al. 2018). The past decades witnessed the great efforts made by scientists to fully dissect the spheres relating with rice production (Chen et al. 2022).

Brown planthopper (BPH) is the most destructive pest to rice, the yield loss caused by BPH in recent years tops the other factors such as fungal and bacterial pathogens in China. Meanwhile, BPH has wing polymorphism distinguished by long winged BPH, and short winged ones. The long-winged BPH are endowed with the ability of long distance migration and thus widespread propagation throughout the rice planting Asia and South–East Asian areas (Xu and Zhang 2017). BPH belongs to the phloem-feeding insects. Sucking juice from the phloem using it stylets, BPH causes the rice plants losing nutrition, photosynthesis decay, leaf yellowing, and even dry-up of large crop areas, a phenotype known as hopperburn (Backus et al. 2005). Therefore, serious damage to the crop caused by BPH results in significant economic loss to rice production almost each year (Kaloshian and Walling 2016). Meanwhile, BPH spreads virus such as ragged-stunt virus and grassy-stunt virus during sucking, which further aggregates the damage situation and yield loss.

Traditional chemical control of BPH causes deleterious consequences to the pests’ natural enemies, and furthermore, pollutes the environments and causes potential harm to human health. Based on the practices in breeding and the theory of insect comprehensive management, exploiting rice endogenous resistance is the most effective and environment-friendly way to control BPH. Accordingly, quite a few BPH resistance quantitative traits loci (QTLs) have been identified, and till this year, about 17 of them have been successfully cloned and mechanism revealed (Shi et al. 2021; Zhou et al. 2021). Utilization of these QTLs effectively directs rice BPH resistance breeding in practice (Sani Haliru et al. 2020; Wang et al. 2016). Rice (and some wild rice) is the only food resource for BPH, during the long co-evolution between rice and BPH, BPH has developed different biotypes and adopted the ability of quick exchange among different biotypes to overcome the resistance variety. Therefore, more endogenous genes are needed to deal with the resistance of the newly emerged biotypes (Sani Haliru et al. 2020). And it would be more ideal if one gene confers broad and durable resistance to more than one biotypes, such as *BPH3*, which confer resistance through clustering of three genes encoding plasma membrane-localized lectin receptor kinases (Liu et al. 2015b), and *BPH6*, which localizes to exocysts and interacts with the exocyst subunit OsEXO70E1 and mediates resistance through increasing exocytosis (Guo et al. 2018).

Meanwhile, concentrated studies from recent years revealed that microRNAs (miRNAs) ranged 21–24 nucleotide (nt) have emerged as important regulators in both plant development and physiology and immunity, with great potential meaning on crop improvement (Song et al. 2019; Tang and Chu 2017). Therefore, miRNAs provides an abundant resource for reverse genetics as a powerful supplement for map-based cloning. Accordingly, miRNAs functioning in rice–BPH interaction have been exploited. In plants with BPH resistance mediated by *BPH15*, a few miRNAs, together with various downstream pathways are differentially expressed compared with those in susceptible plants, and even more miRNAs are differentially expressed upon BPH infestation, indicating activation of miRNAs by BPH feeding (Wu et al. 2017). Indeed, BPH infestation influences expression of many miRNAs, including miR396. Further study revealed that miR396/GRF module regulates rice resistance to BPH through direct transcriptional regulation on the flavonoid biosynthesis pathway, and increasing flavonoid might be a general strategy for plant against BPH (Dai et al. 2019). In addition, miR156 negatively regulates rice resistance to BPH through the jasmonic acid (JA) biosynthetic pathway (Ge et al. 2018).miR159 is one of the most ancient and conserved miRNA among different plant species. Targets of miR159 belongs to the *GAMYB* gene family, which encodes a group of R2R3 MYB domain transcription factors that act as important transducers of gibberellin signals in both seeds and anthers (Aya et al. 2009; Gubler et al. 1995). In consistence with the expression characteristic, miR159 targeting *AtMYB101*, *AtMYB33* and *AtMYB65* to influence vegetative growth, flowering time, anther development and seed size in *Arabidopsis* (Allen et al. 2007; Millar and Gubler 2005; Reichel and Millar 2015). In rice, OsmiR159 positively regulates organ size, including stem, leaf, and grain size through influencing cell division, which further showed the conservation of miR159 in both *Arabidopsis* and rice (Zhao et al. 2017). Further study revealed that OsmiR159–OsGAMYBL2 acts as a common component functioning to connect BR signaling and GA synthetic pathways, and thus modules plant development (Gao et al. 2018). However, whether miR159 function in plant–insect interaction is not clear.

Heterotrimeric G proteins consisting of Gα, Gβ and Gγ subunits are universal signaling modules in eukaryotic organisms that regulate transmembrane signaling by coupling to cell surface-localized receptors (Liang et al. 2018). In *Arabidopsis*, G proteins have been revealed to play an important role in RK-mediated immune signaling (Liang et al. 2018; Liu et al. 2013). Rice genome encodes one each of Gα and Gβ, and five of Gγ (Botella 2012), with most of them functioning in rice grain size regulation (Liu et al. 2018; Sun et al. 2018; Ueguchi-Tanaka et al. 2000; Utsunomiya et al. 2011). Besides, Gγ subunit DEP1 functions in panicle architecture and nitrogen-use efficiency (Huang et al. 2009; Sun et al. 2014), Gγ subunits RGG1 and RGG2 are involved in abiotic stresses regulation (Swain et al. 2017; Yadav et al. 2012). Recently, Gγ subunit GS3 is further proved to mediate rice resistance to high temperature through regulating wax content (Kan et al. 2022). But whether rice G proteins are involved in regulation against BPH is still not clear.

Here we showed that *GS3* gene in rice functioned as a negative regulator in BPH resistance, with knock out of *GS3* plants more resistant to BPH, while over expression of *GS3-4* susceptible to BPH. Meanwhile, OsmiR159 was proved to negatively regulate BPH resistance, while *GAMYBL2* positively regulated BPH resistance. Further genetic study revealed that OsmiR159–OsGAMYBL2 module might function upstream of *GS3* in regulating BPH resistance. OsmiR159 might directly regulate *GS3* gene through direct binding of OsGAMYBL2 to the promoter of *GS3* gene and repressing its expression. Thus, we revealed a new OsmiR159–OsGAMYBL2–GS3 genetic pathway in rice that function in the regulation of BPH resistance.

## OsmiR159 and Target Genes Responded to BPH Infestation

**Fig. 1:**
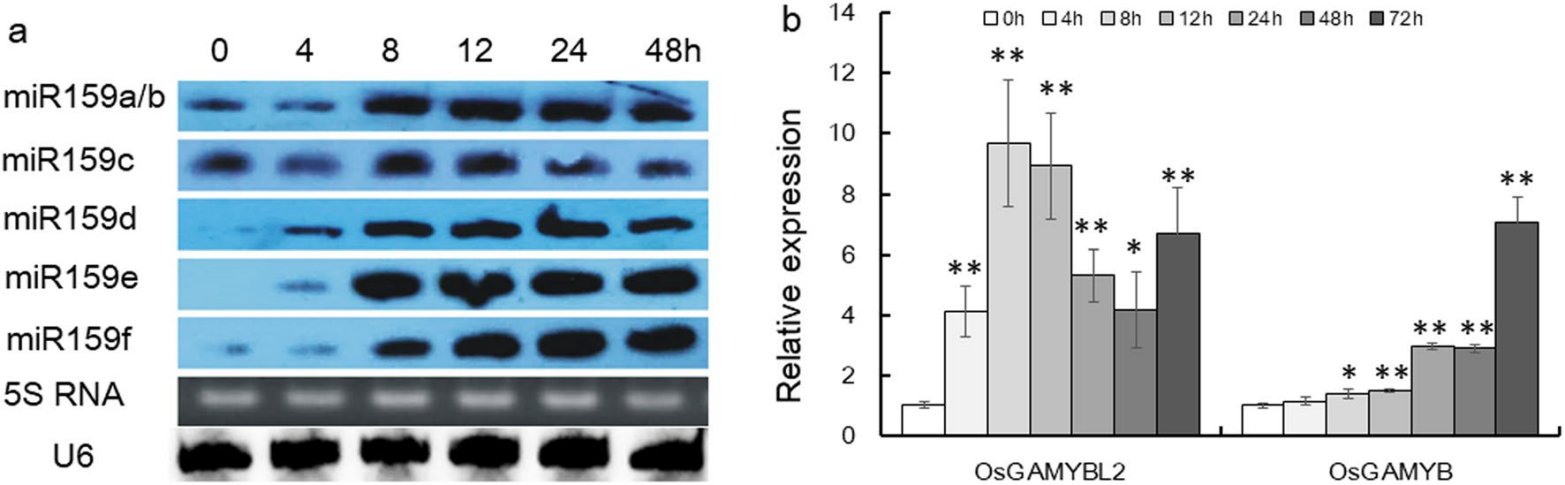
Expression of OsmiR159s and their target genes upon BPH infestation. **a** miRNA Northern detection of different OsmiR159 members at different time after BPH infestation. The sequences of mature miR159a and miR159b are the same. 5S RNA was used as a loading conteol. U6 was used as an internal reference. **b** Detection of *OsGAMYB* and *OsGAMYBL2* mRNA level after BPH infestation using qRT–PCR (n = 3). The expression at “0 h” was set as 1.0, and asterisks indicated significant differences compared with “0 h” as determined by Student’s *t*-test (**P < 0.01; *P < 0.05)

To investigate the possible involvement of OsmiR159 in BPH resistance, we first detected the expression of *OsMIR159* genes upon BPH infestation. There are six genes encoding OsmiR159 in rice (http://structuralbiology.cau.edu.cn/PNRD), with mature OsmiR159a and OsmiR159b sharing the same sequences. We tested *OsMIR159* expression at different time points after BPH infestation by miRNA Northern blot of mature OsmiR159s, it was revealed that all the members of OsmiR159 was greatly up-regulated by BPH infestation (Fig. 1a). Among them, OsmiR159d and OsmiR159e could be up-regulated as early as 4 h post infestation, and most OsmiR159s were fully boasted at 8 h (Fig. 1a).

Next, we wondered if the targets of OsmiR159 could also be influenced by BPH infestation. We detected the expression of *OsGAMYB* and *OsGAMYBL2*, two target genes of OsmiR159 (http://structuralbiology.cau.edu.cn/PNRD), by qRT–PCR. It was revealed that both genes were up-regulated by BPH infestation, with induction of *OsGAMYBL2* more obvious and rapid than that of *OsGAMYB* (Fig. 1b).

Therefore, from these assays, it was clear that OsmiR159s and their target genes were responsive to BPH infestation, indicating possible involvement in BPH resistance.

## OsmiR159 Negatively Regulated BPH Resistance

**Fig. 2:**
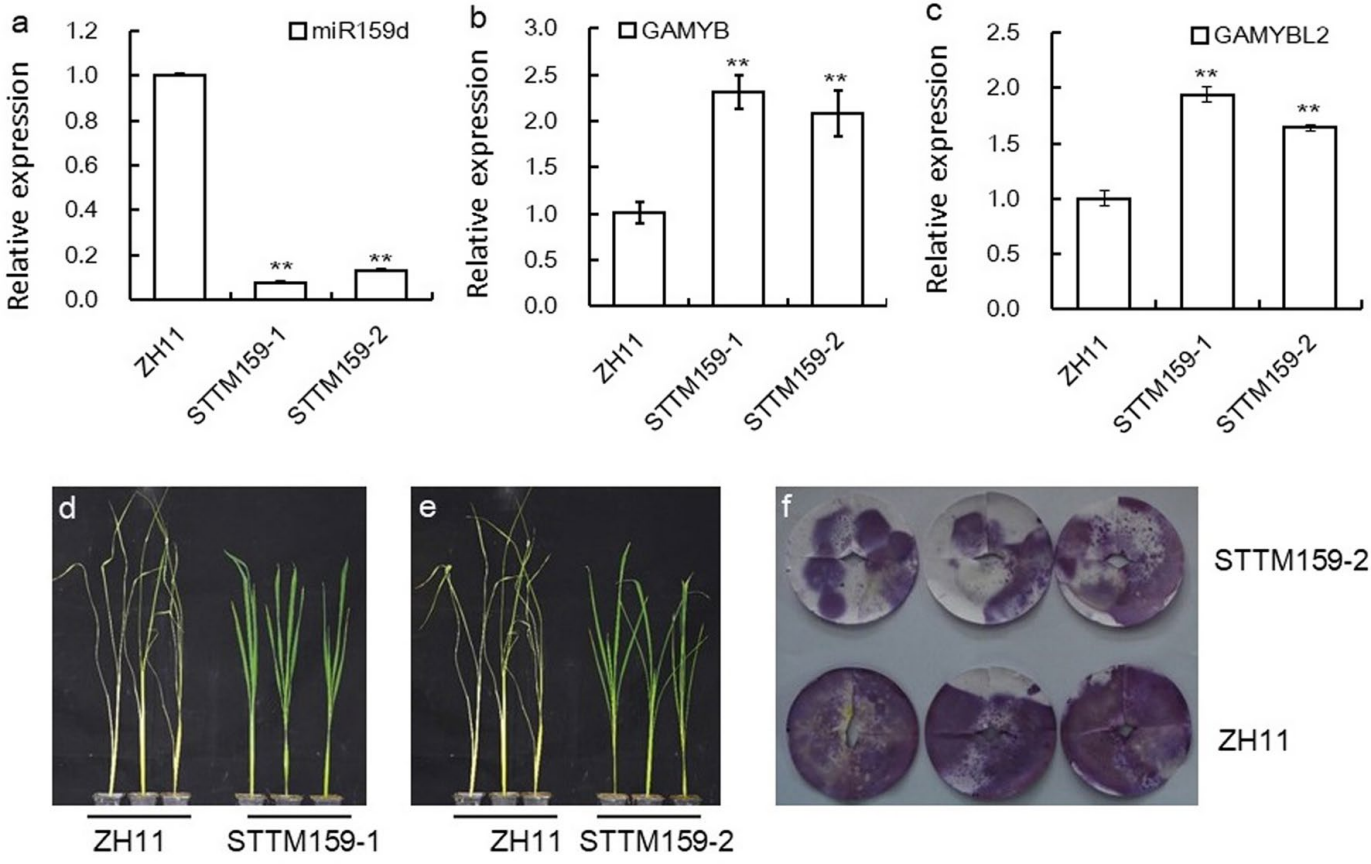
Detection of STTM159 plants against BPH. **a** Expression of OsmiR159d in STTM159 and WT plants revealed by stem-loop qRT–PCR (n = 3). **b** Expression of *OsGAMYB* gene in STTM159 and WT plants revealed by qRT–PCR (n = 3). **c** Expression of *OsGAMYBL2* gene in STTM159 and WT plants revealed by qRT–PCR (n = 3). **d**, **e** The plant status of STTM159-1, STTM159-2 and control ZH11 plants after BPH infestation for 11 days in an individual test assay. **f** Honeydew display of the BPH after feeding on STTM159-2 and WT plants. Asterisks in **a**–**c** indicated significant differences compared with ZH11 plants as determined by Student’s *t*-test (**P < 0.01), with the expression level in ZH11 was set as 1.0

Now that OsmiR159s and their target genes could be obviously up-regulated by BPH infestation, we next detected the genetic function of OsmiR159 against BPH. The STTM159 plants we used were previously reported to mediate the interaction between GA and BR pathway in rice (Gao et al. 2018). To test if OsmiR159 function in BPH resistance, we carried out individual test of two lines of STTM159 plants and the corresponding wild type (WT) ZH11. Beforehand, we carried out stem-loop qRT–PCR and verified that using OsmiR159d as an example, OsmiR159 in these two lines was significantly decreased (Fig. 2a). While accordingly, *OsGAMYB* and *OsGAMYBL2* gene was obviously up-regulated (Fig. 2b, c). Then in individual test, it was revealed that when ZH11 plants died around seven days after BPH infestation, the two lines of STTM159 plants were still alive (Fig. 2d, e), indicating that STTM159 plants were more resistance to BPH than the WT. Meanwhile, we got the STTM159 plants in NIP background (named STTM159n hereafter) (Zhao et al. 2016), the miR159d was obviously down-regulated in STTM159n plants (Additional file 1: Fig. S1a). We then detected the response of them to BPH using individual test, it was revealed that STTM159n plants also died later than their WT NIP (Additional file 1: Fig. S1b), further confirming that OsmiR159 negatively regulated rice resistance to BPH.

Next, we checked the resistance mechanism of the STTM159 plants. Usually, plant takes three kinds of mechanism in resistance to insect herbivory, antixenosis to affect insect settling, colonization, and oviposition; antibiosis to reduce insect survival rates or feeding activity; and tolerance to withstand insect damage (Nalam et al. 2019). We checked the honeydew, the excreta of BPH when feeding, which can comparably reflect the amount of intake. It was revealed that BPH produced much less honeydew when feeding on STTM159 plants, while much more honeydew was produced when they feed on the WT plants (Fig. 2f). Therefore, STTM159 plants might take an antibiotic mechanism to resist BPH.

**Fig. 3:**
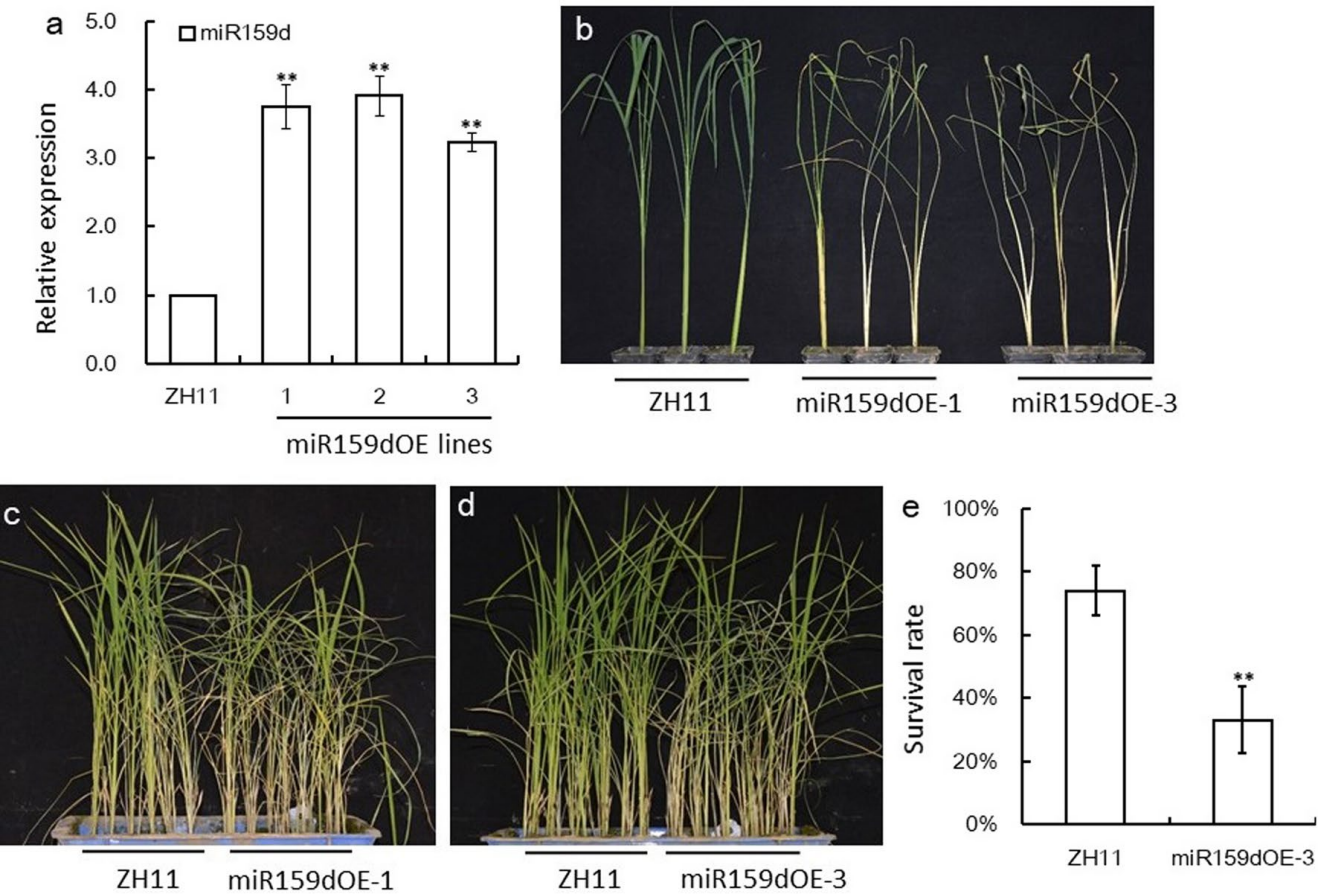
Verification of miR159dOE plants and BPH resistance test. **a** Expression of OsmiR159d in miR159dOE and WT plants revealed by stem-loop qRT–PCR (n = 3). The expression level in ZH11 was set as 1.0, and asterisks indicated significant differences compared with ZH11 plants as determined by Student’s *t*-test (**P < 0.01). **b** The plant status of miR159dOE-1, miR159dOE-3 and control ZH11 plants after BPH infestation for 11 days in an individual test assay. **c**, **d** The plant status of miR159dOE-1 and miR159dOE-3 plants verse WT plants after BPH infestation for about 9 days in a mall population test. **e** Survival rate of the plants in small population test in (**d**). Values are given as means +/− SDs (n = 3). Asterisks indicated significant differences compared with ZH11 plants as determined by Student’s *t*-test (**, *P* < 0.01)

Furthermore, we constructed the miR159dOE plants in ZH11 genetic background. Over expression of OsmiR159d was verified by stem-loop qRT–PCR (Fig. 3a), and accordingly the GAMYBL2 gene was down-regualted in these lines (Additional file 1: Fig. S2). We first took two lines of miR159dOE plants, miR159dOE-1 and miR159dOE-3, to check the BPH resistance of them using individual test, it was revealed that both lines of miR159dOE plants died earlier than WT after BPH infestation (Fig. 3b). Then we used small population test to check BPH resistance of miR159dOE-1 and miR159dOE-3 plants. Both lines died earlier than their respective WT ZH11 controls (Fig. 3c, d), and the survival rate of the miR159dOE-3 was much lower than that of the WT (Fig. 3e), indicating that miR159dOE lines were susceptible to BPH infestation.

## *OsGAMYBL2* Genes Positively Regulated BPH Resistance

**Fig. 4:**
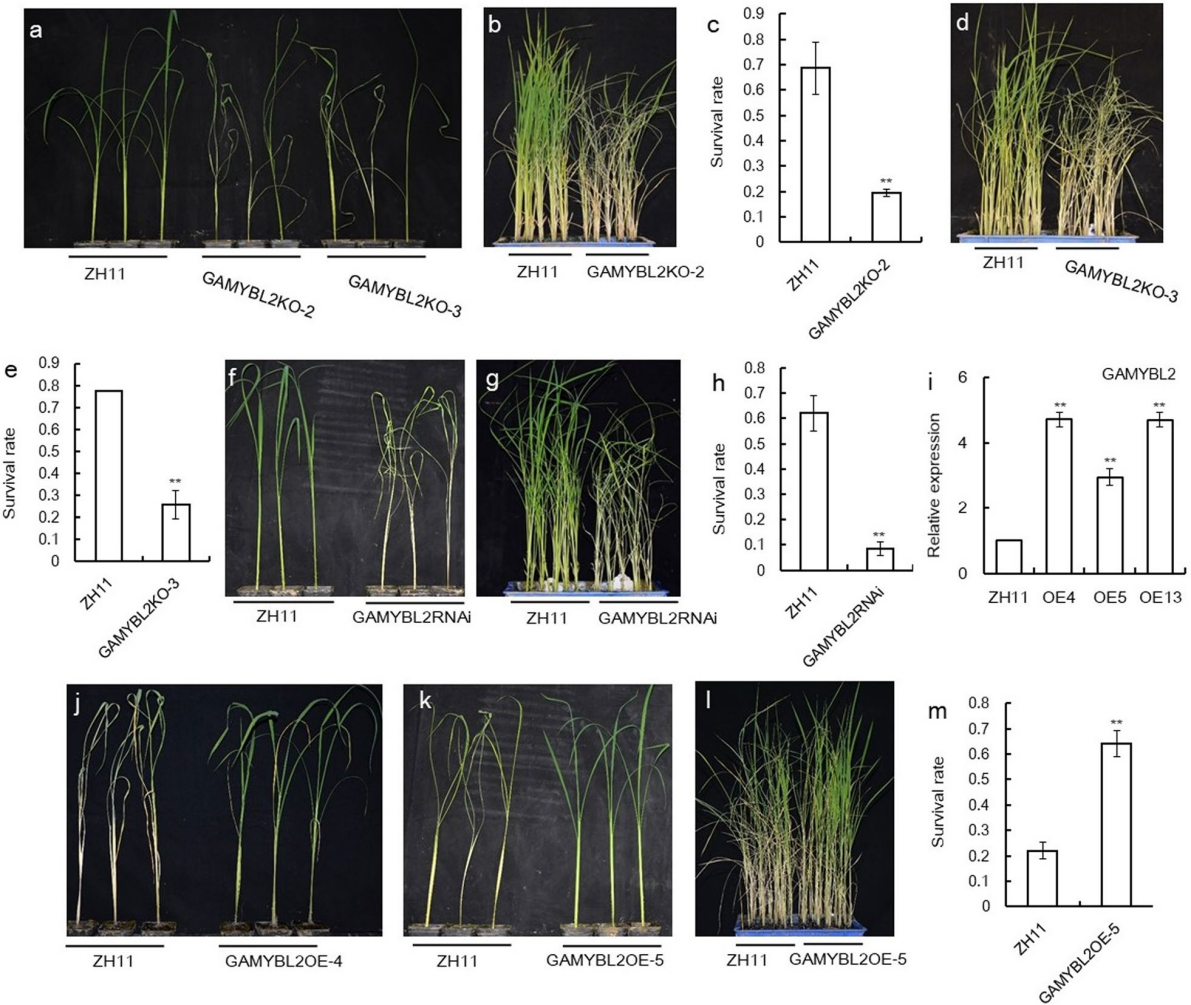
BPH resistance test of GAMYBL2RNAi and GAMYBL2KO plants. **a** The plant status of GAMYBL2KO-2, GAMYBL2KO-3 and control ZH11 plants after BPH infestation for 8 days in an individual test. **b** The status of the GAMYBL2KO-2 plants and WT plants after BPH infestation for 12 days in a small population test. **c** Survival rates of the plants in **b**. **d** The status of the GAMYBL2KO-3 plants and WT plants after BPH infestation for about 10 days in a small population test. **e** Survival rates of the plants in **d**. **f** The plant status of GAMYBL2RNAi and ZH11 plants after BPH infestation for 7 days in an individual test. **g** The plant status of GAMYBL2RNAi and ZH11 plants after BPH infestation for about 5 days in a small population test. **h** Survival rate of the plants in small population test In **g**. **i** Expression of *OsGAMYBL2* gene in GAMYBL2OE and WT plants revealed by qRT–PCR (n = 3). Asterisks in indicated significant differences compared with ZH11 plants as determined by Student’s *t*-test (**P < 0.01), with the expression level in ZH11 was set as 1.0. **j** The plant status of GAMYBL2OE-4 and ZH11 plants after BPH infestation for 7 days in an individual test. **k** The plant status of GAMYBL2OE-5 and ZH11 plants after BPH infestation for 12 days in an individual test. **l** The status of the GAMYBL2OE-5 plants and WT plants after BPH infestation for about 10 days in a small population test. **m** Survival rates of the plants in **l**. In figures **c**, **e**, **h** and **m**, values are given as means ± SDs (n = 3). Asterisks indicated significant differences compared with ZH11 plants as determined by Student’s *t*-test (**P < 0.01)

Since *OsGAMYBL2* responded to BPH infestation very obviously (Fig. 1b), we deduced that it might function in BPH resistance. To explore the genetic function of *OsGAMYBL2* in BPH resistance, we made *OsGAMYBL2* gene edited plants using CRISPR-Cas9 technology with a gDNA designed close to the ATG start code, the plants got were named *GAMYBL2KO* plants. In the T0 generation, we got 4 lines of homologous edited plants (Additional file 1: Fig. S3a). We carried out BPH resistance analysis of the GAMYBL2OE plants and GAMYBL2KO plants. In individual test, two lines of GAMYBL2KO plants, GAMYBL2KO-2 and GAMYBL2KO-3 both died earlier than ZH11 (Fig. 4a), indicating that they were much susceptible to BPH infestation. And then in small population test, also, both lines of GAMYBL2KO plants died earlier than their respective WT ZH11 plants (Fig. 4b, d); furthermore, statistical analysis of the survival rates of GAMYBL2KO-2 and GAMYBL2KO-3 plants were respectively lower than that of the WT (Fig. 4c, e). In addition, we carried out small population test using the GAMYBL2RNAi plants (Gao et al. 2018), in which *OsGAMYBL2* gene was down-regulated (Additional file 1: Fig. S3b). It was revealed that the GAMYBL2RNAi plants died earlier than the WT ZH11 either in both individual test or small population test (Fig. 4f, g), with the survival rate of the GAMYBL2RNAi plants much lower than that of ZH11 plants (Fig. 4h).

Meanwhile, we made *OsGAMYBL2* gene over expression plants under drive of the 35S promoter, the plants got were named GAMYBL2OE. In the GAMYBL2OE transgenic plants, over expression of *OsGAMYBL2* gene was verified by qRT–PCR (Fig. 4i). Then we used two lines of GAMYBL2OE, GAMYBL2OE-4 and GAMYBL2OE-5, to do individual test, it was revealed that both lines died later than WT after BPH infestation (Fig. 4j, k). In a small population test, the GAMYBL2OE-5 plants also died later than WT (Fig. 4l), with the survival rate much higher than the WT (Fig. 4m). Altogether, from these assays, it was clear that *OsGAMYBL2* positively regulated BPH resistance.

## OsGAMYBL2 Directly Regulated *GS3* Gene at Transcriptional Level

**Fig. 5:**
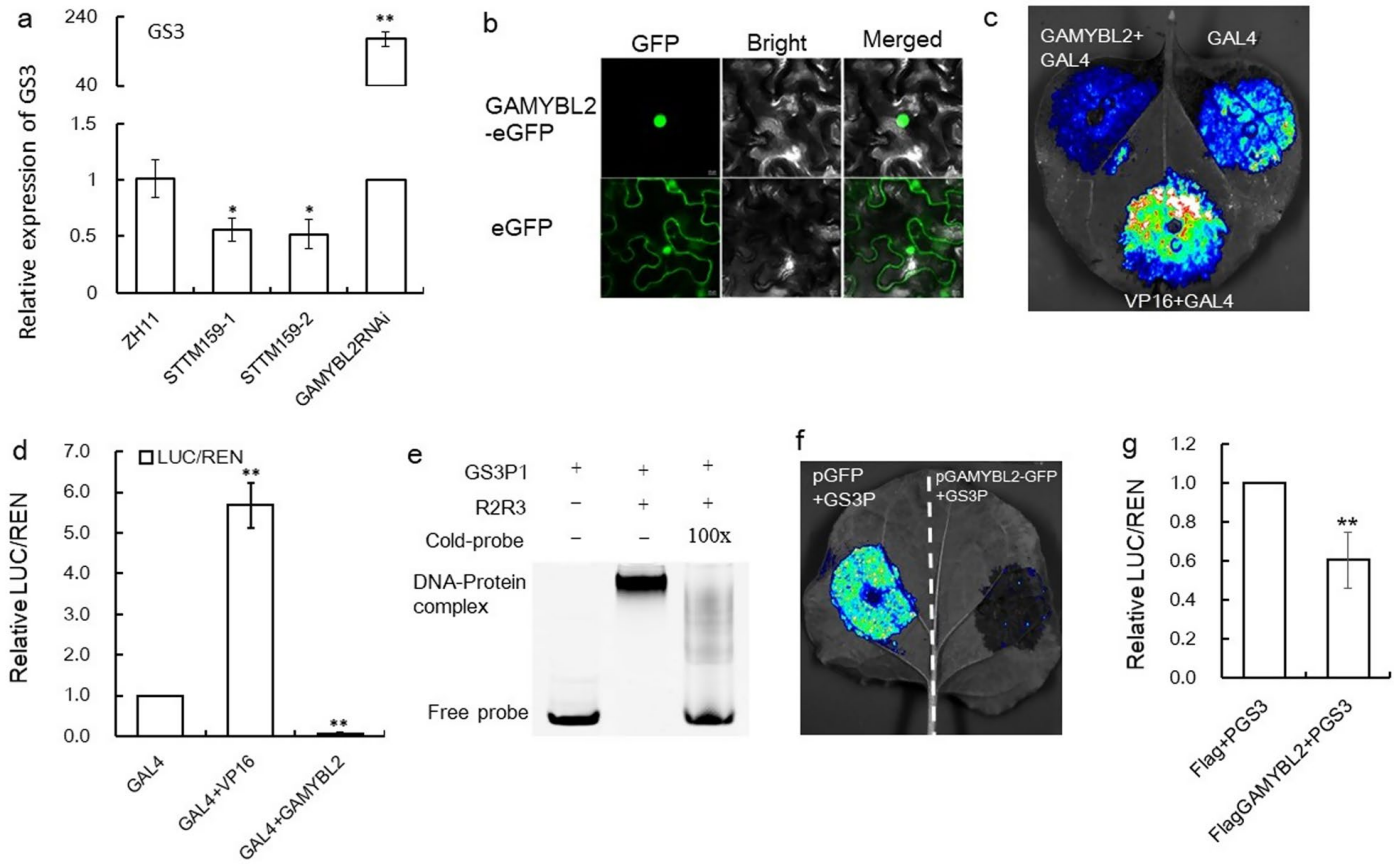
Molecular and biochemistry assays proving the transcriptional regulation of OsGAMYBL2 on *GS3* gene. **a** Expression of *GS3* gene in the STTM159 plants, GAMYBL2RNAi plants and ZH11 plants revealed by qRT–PCR (n = 3). The expression level in ZH11 was set as 1.0, and asterisks indicate significant differences compared with ZH11 plants as determined by Student’s *t*-test (**P < 0.01; *P < 0.05). **b** Sub-cellular localization of OsGAMYBL2 proteins in leaves of Nicotiana Benthamiana. Bar = 10 µm. **c** Luciferase strength of the transient coexpression of the effector and reporter constructs in tobacco leaves. **d** Measurement of the relative LUC/REN ratio after transient coexpression of the effector and reporter constructs in tobacco leaves. The values are means ± SDs (n = 3). The data were normalized to a value of 1 for the GAL4 DBD group. Asterisks indicate significant differences determined by Student’s *t*-test (**, *P* < 0.01). **e** EMSA assay of GAMYBL2 proteins and the promoter fragment of *GS3* gene. **f** Image of Dual-LUC assay of GAMYBL2–GFP fused protein and *GS3* gene promoter showing infloerescence strength. **g** The enzyme activity assay showing LUC/REN ratio of the experiments in E. The ratio in “Flag + pGS3” was set as 1.0, and asterisks indicate significant differences compared with “Flag + pGS3” as determined by Student’s *t*-test (**P < 0.01). Values are given as means ± SDs (n = 3)

In development, miR159/GAMYBL2 and *GS3* both regulate plant architecture and grain size, with STTM159, GAMYBL2OE and GS3-4OE showing similar dwarf plants and small grain size (Gao et al. 2018; Sun et al. 2018). Therefore, we deduced that miR159/GAMYBL2OE and *GS3* might have genetic function through direct transcriptional regulation of GAMYBL2OE on *GS3* gene. To verify, we first detected the expression of *GS3* gene in STTM159 and GAMYBL2RNAi plants, it was revealed that in STTM159 plants, *GS3* gene was obviously down-regulated, while in the GAMYBL2RNAi plants, it was obviously up-regulated (Fig. 5a), indicating that *GS3* gene might function downstream of OsmiR159 in functioning, and OsGAMYBL2 might inhibit *GS3* gene.

Most target genes of miRNA encode transcriptional factors (Llave et al. 2002). To check the character of OsGAMYBL2 protein, we first tested its cellular localization in leaves of *Nicotiana Benthamiana*, it was revealed that OsGAMYBL2–GFP proteins localized specifically in the nuclear, in contrast, the GFP itself showed a signal of ubiquitous expression (Fig. 5b), implying that OsGAMYBL2 protein localized in the nuclear, and had the possibility to be a transcriptional factor. Next, we analyzed the transcriptional activation/repression of the OsGAMYBL2 protein using the GAL4/UAS system. We took the GAL4 DNA-binding domain (GAL4-DBD), GAL4-DBD-OsGAMYBL2 and GAL4-DBD-VP16 as effectors for expression comparision (Additional file 1: Fig. S4). It was revealed that that GAL4 DBD-VP16 showed a higher luciferase strength than GAL4-DBD, while GAL4-DBD-OsGAMYBL2 showed a lower luciferase strength than GAL4-DBD (Fig. 5c). According, the relative firefly luciferase/Renilla luciferase (LUC/REN) ratio further verified this result (Fig. 5d), indicating OsGAMYBL2 might be repressor.

To verify if OsGAMYBL2 protein could bind to the promoter of *GS3*, we first analyzed the putative binding motif for OsGAMYBL2 protein. We first consulted the binding motif of AtMYB101, the homolog with the highest homology to OsGAMYBL2 in Arabidopsis, on the JASPAR website (https://jaspar.genereg.net/) to be T(C/A)AACNG(A) (Additional file 1: Fig. S5a). And then we used EMSA and revealed that the DNA-binding domain of OsGAMYBL2 (GL2R2R3) could bind to the predicted TAACCG motifs (Additional file 1: Fig. S5b), when TAACCG motifs were mutanted to “GGGTCG”, the binding vanished (Additional file 1: Fig. S5c). Accordingly, we found 8 OsGAMYBL2 binding motifs in the 2 Kb *GS3* promoter upstream of the starting code ATG (Additional file 1: Fig. S6). Next, we used EMSA and verified that OsGAMYBL2 protein could bind to the promoter of *GS3* gene (Fig. 5e). Furthermore, in Dual-LUC assay, the GAMYBL2-GFP and *GS3* promoter produced no florescence signal, while that of GFP void plasmid and *GS3* give florescence signal (Fig. 5f), enzyme activity assay of LUC and REN from Dual-LUC further verified obvious difference of GAMYBL2–GFP and control GFP in activating *GS3* promoter (Fig. 5g). These assays indicated that OsGAMYBL2 protein might bind to the promoter of *GS3*, and so that inhibit the expression of *GS3* gene.

## *GS3* gene Negatively Regulated BPH Resistance

**Fig. 6:**
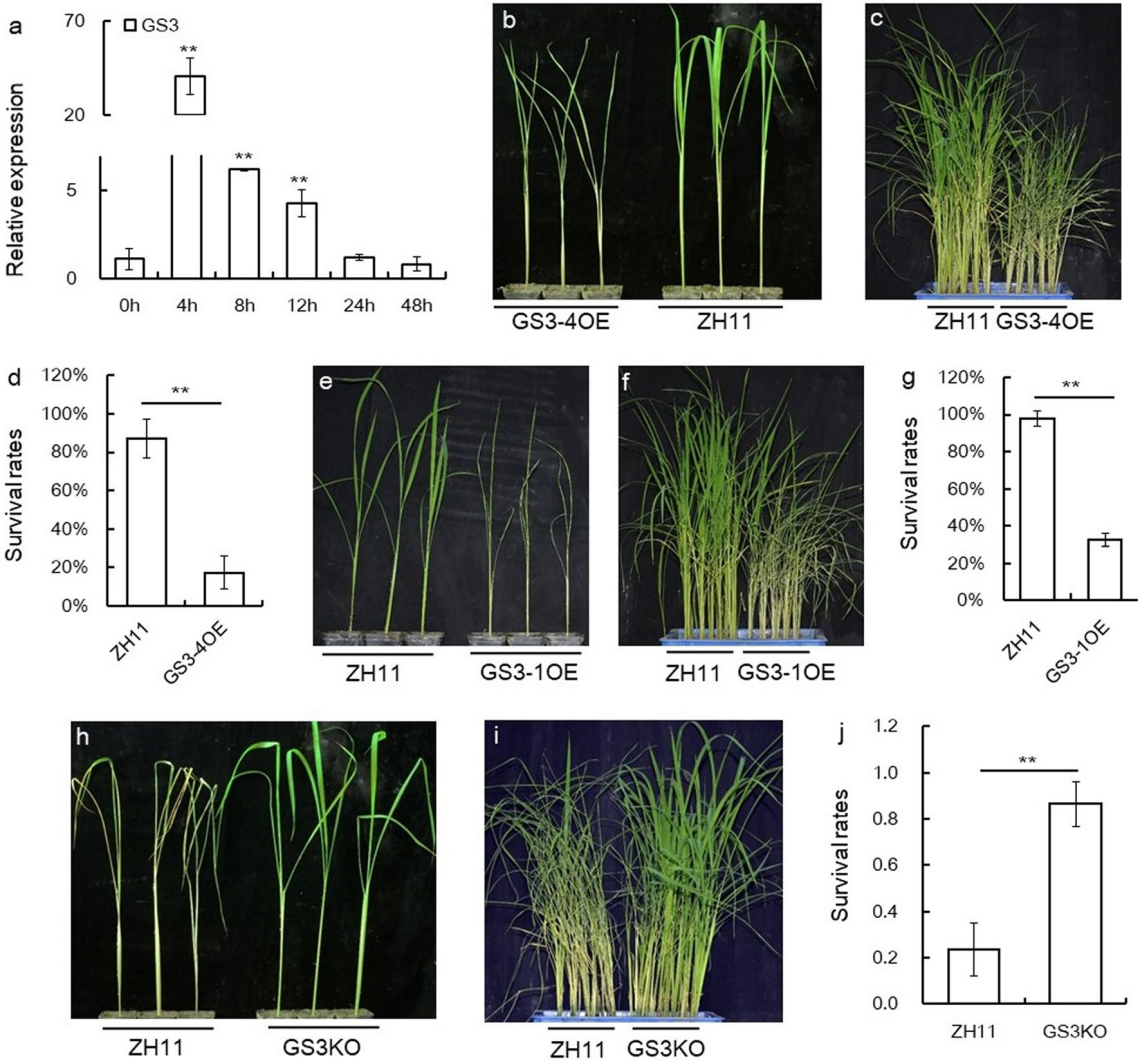
Expression of *GS3* gene and BPH resistance test of GS3OE and GS3KO plants. **a** Expression of *GS3* gene in response to BPH infestation. The expression level at 0 h was set as 1.0, and asterisks indicate significant differences comparing with 0 h as determined by Student’s *t*-test (**P < 0.01). **b** The plant status of GS3–4OE and ZH11 plants after BPH infestation for 9 days in an individual test. **c** The plant status of GS3–4OE and ZH11 plants after BPH infestation for about 5 days in a mall population test. **d** Survival rates of the plants in **c**. **e** The plant status of GS3–1OE and ZH11 plants after BPH infestation for 4 days in an individual test. **f** The plant status of GS3–1OE and ZH11 plants after BPH infestation for about 5 days in a mall population test. **g** Survival rates of the plants in **f**. **h** The plant status of GS3KO and ZH11 plants after BPH infestation for 12 days in an individual test. **i** The plant status of GS3KO and ZH11 plants after BPH infestation for about 5 days in a mall population test. **j** Survival rates of the plants in **i**. Asterisks in **d**, **g**, **j** indicate significant differences as determined by Student’s *t*-test (**P < 0.01)

Now that *GS3* gene was transcriptionally regulated by GAMYBL2, we wonder if *GS3* gene function in BPH resistance. We first checked the expression of *GS3* gene upon BPH infestation, it was revealed that *GS3* gene was induced by BPH infestation obviously and promptly (Fig. 6a), indicating *GS3* gene is a quick responsive gene to BPH attack. Next, we used the GS3-1OE, GS3-4OE plants and GS3KO plants for BPH resistance test (Sun et al. 2018). In both the GS3-1OE and GS3-4OE plants, GS3 gene was obviously up-regualted as compared with in the WT (Additional file 1: Fig. S7a). And in the GS3KO plants, the GS3 gene was edited (Additional file 1: Fig. S7b). In both individual test and small population test, the GS3-4OE plants died earlier than the corresponding ZH11 plants (Figs. 6b, c), and the survival rate of the GS3-4OE plants was lower than that of ZH11 in small population test (Fig. 6d). Also, the GS3-1OE plants died earlier than ZH11 whether in individual test or small population test (Figs. 6e, f), and the survival rate of the GS3-1OE plants was obviously lower than that of ZH11 in small population test (Fig. 6g). To the contrary, GS3KO plants died later than ZH11 whether in individual test or small population test (Figs. 6h, i), and the survival rate of GS3KO plants was much higher than that of ZH11 in small population test (Fig. 6j).

Furthermore, in an small population test when GS3-4OE plants, GS3KO plants and WT plants were grown together, after BPH infestation, the GS3-1OE plants died the earliest (Additional file 1: Fig. S8a), and then along with the feeding progressed, the ZH11 plants died (Additional file 1: Fig. S8b), while the GS3KO plants were still alive till several days later. From these assays, it is clear that *GS3* gene negatively regulated BPH resistance.

## Cellulose Synthesis Genes Might Function Downstream *GS3* to Mediate BPH Resistance

**Fig. 7:**
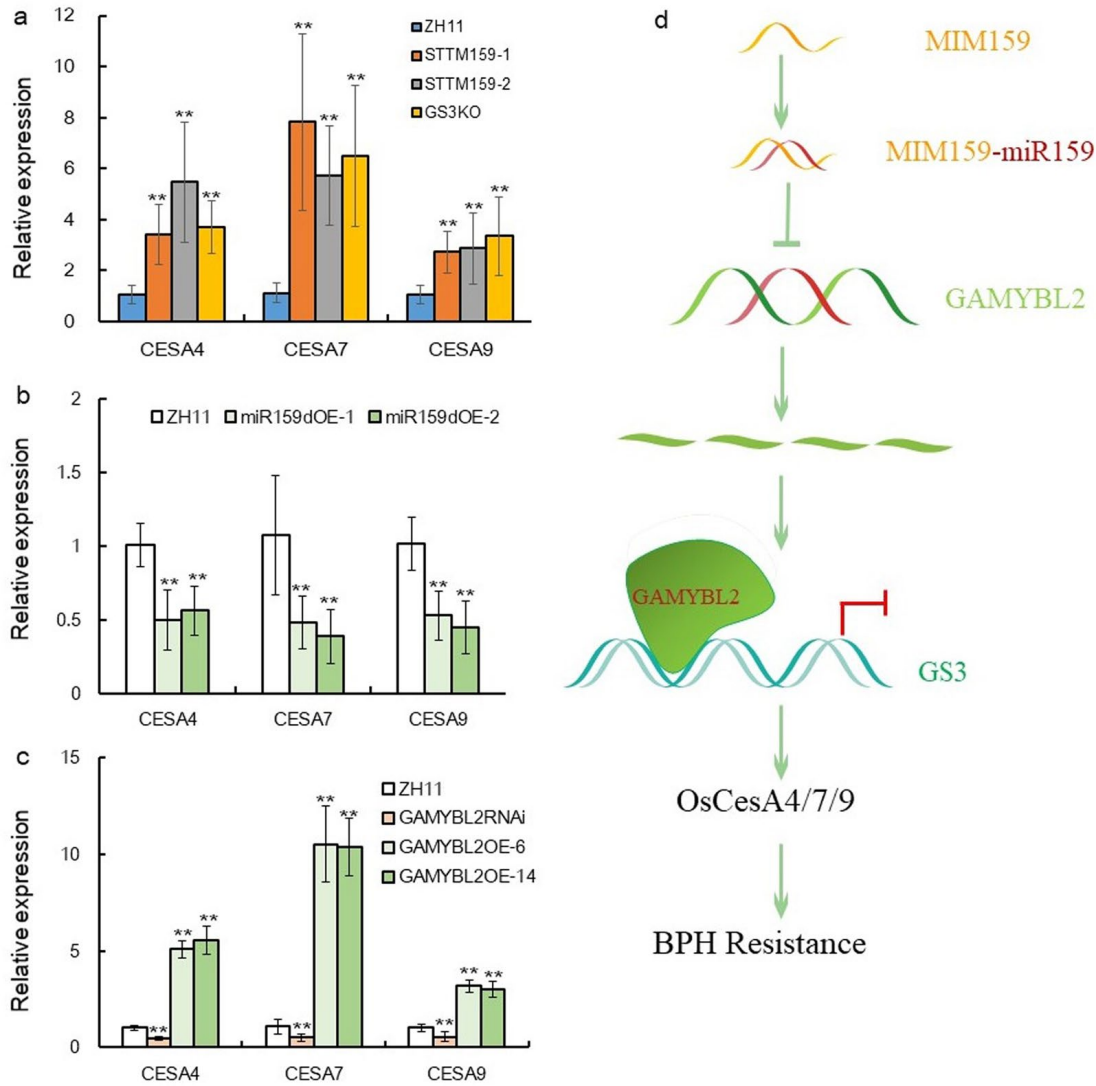
Expression of cellulose synthesis genes and sketch map of the mechanism underlying the OsmiR159–GAMYBL2–GS3 pathway against BPH. **a** Expression of cellulose synthesis genes in STTM159, GS3KO and ZH11 plants. **b** Expression of cellulose synthesis genes in miR159dOE and ZH11 plants. **c** Expression of cellulose synthesis genes in GAMYBL2OE, GAMYBL2RNAi and ZH11 plants. **a**–**c** The expression level in ZH11 was set as 1.0, and asterisks indicate significant differences comparing with that in ZH11 as determined by Student’s *t*-test (**P < 0.01). **d** A sketch map illustrating the mechanism of the OsmiR159-GAMYBL2-GS3 pathway. Mimicry OsmiR159 inhibits the function of OsmiR159, thus release its inhibition on *GAMYBL2* mRNA, elevated GAMYBL2 proteins repressed the expression of *GS3* gene, and thus rendered resistance to BPH through promoting the expression of cellulose synthesis genes such as *OsCesA4*, *OsCesA4*, *OsCesA4*

In rice, the BPH resistance gene BPH30 increases the expression of cellulose and hemicellulose synthesis genes and makes the cell walls stiffer and sclerenchyma thicker, increasing the resistance to BPH (Shi et al. 2021). We wonder if cellulose synthesis genes also fuction in the OsmiR159–OsGAMYBL2–GS3 pathway, it was revealed that expression of cellulose synthesis genes such as *OsCESA4*, *OSCESA7*, *OsCESA9* (Tanaka et al. 2003) were all up-regulated in the STTM159, GAMYBL2OE plants and GS3KO plants, while in miR159dOE and GAMYBL2RNAi plants, these genes were obvioudly down-regualted as compared with that in ZH11 (Figs. 7a–c). Therefore, these genes might function downstream of *GS3* to mediate BPH resistance, accordingly, the mechanism of the OsmiR159–OsGAMYBL2–GS3 modulating BPH resistance was summarized in Fig. 7d.

Furthermore, we checked the expression of *GAMYBL2* and *GS3* in the STTM159 plants before and after BPH infestation. As shown in Additional file 1: Fig. S9a, in STTM159, *GAMYBL2* gene was induced at 8 h after BPH infestation, while in ZH11, it was up-reuglaed at 4 h (Fig. 1b). Also in STTM159, *GS3* was induced at 8 h after BPH infestation (Additional file 1: Fig. S9a), while in ZH11, it was up-regulated at 4 h (Fig. 6a). Therefore, the induction of both *GAMYBL2* and *GS3* in the STTM159 plants by BPH was abated, the abation of *GS3* was especially obviously, indicating that in the OsmiR159–OsGAMYBL2–GS3 pathway, up-regulation of *GAMYBL2* in STTM159 further suppressed or retarded the expression of *GS3* especially upon BPH infestation.

## Discussion

For the first time, we reported that G proteins function in rice resistance against BPH, a kind of piercing and sucking insect pest. As important factors in signal transduction, heterotrimeric G proteins play important roles in multiple physiological processes in both animal and plants. In plant, G proteins signaling not only modulate plant growth and development, but also influence biotic and abiotic stress response (Zhang et al., 2021). G proteins function in various abiotic stresses such as drought, salt, low and high temperature. In rice, RGA1 negatively regulated drought stress (Ferrero-Serrano and Assmann 2016), the Gβ subunit RGB1 and Gγ subunit qPE9-1 are also involved in the response to ABA signaling and drought stress (Zhang et al. 2015). Furthermore, RGA1 mediates cold response in association with Chilling Tolerance Divergence1 (COLD1) to activate Ca^2+^ channel and enhance the GTPase activity of the G protein (Ma et al. 2015). Interestingly, *GS3* was recently identified as a QTLs that negatively mediate high temperature through influencing wax synthesis (Kan et al. 2022). It has been suggested that G proteins mediate plant immunity through regulating programmed cell death and stomatal closure (Zhang et al. 2012), the underlying molecular mechanism was advanced by discovering receptor-like kinases (RLKs) as the corresponding G protein-coupled receptors (GPCRs) in animal, which was missing in plants (Tunc-Ozdemir and Jones 2017; Zhong et al. 2019). The heterotrimeric Gβγ subunits, AGB1 and AGG1/AGG2, act downstream of multiple RLKs to activate plant resistance to pathogens in *Arabidopsis* (Liu et al. 2013). RGS1, a GTPase accelerating protein that regulated by multiple immune receptors, regulates G proteins inactive/active state in complex with FLS2 through phosphorylation (Liang et al. 2018). Nevertheless, no G proteins have been reported to function in plant resistance against insects, here we showed that *GS3* gene promptly responded to BPH attack (Fig. 5a), and negatively regulated rice resistance to BPH (Figs. 5b–d), indicating that besides regulating grain size (Sun et al. 2018), high temperature response (Kan et al. 2022), *GS3* gene also mediates biotic response in rice, and this might function through influencing cellulose synthesis, since cellulose synthesis genes such as *CESA4, CESA7, CESA9* was all up-reguated in the STTM159, GAMYBL2OE and GS3KO plants, while down-reugaled in the miR159dOE and GAMYBL2RNAi plants (Figs. 7a–c).

Furthermore, through expression analysis (Fig. 4a), EMSA assay (Fig. 4c) and Dual-LUC assay (Figs. 4d, e), we proved that *GS3* was directly regulated by OsmiR159–OsGAMYBL2 at the transcriptional level, OsGAMYBL2 might function as a transcriptional factor that repress the function of *GS3* in BPH resistance. In higher plants, MYB proteins regulate early metabolism through formation of MYB/bHLH/WD40 (MBW) complexes to module development and environmental response (Liu et al. 2015a; Xu et al. 2015). Specifically, *OsMYB30* positively regulate BPH resistance through direct regulation of *phenylalanine ammonia-lyase6* (*OsPAL6*) and *OsPAL8* gene, which participate in the phenylpropanoid biosynthesis pathway and positively regulate BPH resistance by influencing lignin and SA contents (He et al. 2020). In this study, we identified another MYB protein, GAMYBL2 to positively regulate BPH resistance (Fig. 3), indicating possible active participation of MYB proteins in BPH response, whether other MYB proteins function in BPH resistance deserves further investigation.miRNA and its targets usually function not only in plant development, but also in plant physiology. For example, miR156 and SPL genes not only regulate plant architecture, but also mediate immunity to pathogen and BPH (Dai et al. 2018; Ge et al. 2018; Liu et al. 2019a). miR396 and target genes regulate grain size and panicle architecture in development, and meanwhile modulate BPH resistance (Dai et al. 2019; Gao et al. 2015; Yang et al. 2021). miR319 and target TCP genes not only regulate leaf size, but also mediate plant resistance to salt and immunity (Liu et al. 2019b; Yang et al. 2013; Zhang et al. 2018). miR159 is one of the most conserved miRNAs in plants, which has been identified in green algae and liverwort, as long as flowering plants with high abundance (Alaba et al. 2015; Chavez Montes et al. 2014). It has been proved that OsmiR159 and target OsGAMYB genes regulate plant configuration and grain size (Gao et al. 2018). In this study, we further proved that OsmiR159 respond to BPH infestation obviously (Fig. 1a), although there might be cross-hybridization due to the sequence similarity between different members of the OsmiR159 (Chen et al. 2005), the overall trend of induction by BPH feeding is still obvious. And genetic function assay revealed that OsmiR159 negatively regulated BPH resistance, with STTM159 plants showing resistance to BPH and miR159dOE plants showing susceptible to BPH (Fig. 2). Furthermore, we found that OsmiR159 negatively regulated rice drought resistance (date to be published elsewhere). On the other hand, in development, STTM159 plants showed stunt plant architecture and small grain size (Gao et al. 2018), indicating OsmiR159 positively regulate plant growth. We deduced that STTM159 plants function in biotic and abiotic resistance through sacrificing growth, the mechanism through which OsmiR159 balancing growth and resistance deserves further study.miRNAs mainly function by targeting and inhibiting the function of their target genes. In this study, we found that members of OsmiR159 were induced by BPH feeding (Fig. 1a), and*OsGAMYB* and *OsGAMYBL2* were also induced, but not inhibited (Fig. 1b). This inconsistence was also obversed in our previous study, we found that expression of OsmiR396 and some of its target genes were both induced by BPH infestation (Dai et al. 2019). In the case of miRNA function against blast fungus, although many miRNA-target pairs displayed negative correlation between the abundance of miRNAs and their targets, some did not (Li et al. 2013). *OsSPL10* negatively regulated drought toleranc, however, expression of *OsSPL10* is also induced by drought (Li et al. 2022). Therefore, the response of miRNA and targets against adverse stresses are not necessarily always negative, although the underlying mechanism needs further investigation. Furthermore, participation of miRNA in BPH resistance might be more complicated than that in fungus resistance.

In conclusion, we revealed new functions for both OsmiR159–OsGAMYBL2 module and *GS3* gene in BPH resistance, besides their respective function in development control physiological regulation, indicating the multifunctional character of not only OsmiR159–OsGAMYBL2 module, but also G protein encoding *GS3* gene. Furthermore, we proved direct transcriptional regulation of OsGAMYBL2 proteins to *GS3* gene and established a new OsmiR159–OsGAMYBL2–GS3 signaling pathway that mediated BPH resistance in rice.

## Material and Methods

### Plant Species and Growth Conditions

The wild type (WT) rice plants used in this study were varieties ZH11 (*Oryza sativa* L. subsp. *japonica* cv. Zhonghua No.11, ZH11), NIP (*Orayza sativ*a L. subsp. Japonica. cv. Nippobare, NIP), and TN1 (*Oryza sativa* L. subsp. *indica* cv. Taichung Native 1, TN1). All rice plants were cultivated under field conditions at two different experimental stations in Shanghai (30° N, 121° E) and Lingshui (Hainan Province, 18° N, 110° E), China. Rice seedlings were cultures in the phytotron in CAS Center for Excellence in Molecular Plant Sciences, with 30/24 ± 1℃ day/night temperature, 50–70% relative humidity and a light/dark period of 14 h/10 h was used to culture rice seedlings.

GS3-1OE, GS3-4OE and GS3KO plants were kindly presented by Sun et al. (Sun et al. 2018). STTM159 plants and GAMYBL2RNAi plants in ZH11 background were kindly gifted by professor Xiong (Gao et al. 2018). STTM159 plants in NIP background were kindly presented by professor Peng (Zhao et al. 2016).

### BPH Population

The BPH population was originally obtained from rice fields in Shanghai, China, and maintained on susceptible rice variety TN1 in a climate-controlled room at 26 ± 2 °C, 12 h/12 h light/dark cycle and 80% relative humidity.

### Plasmid Construction and Plant Transformation

For *GAMYBL2* over expression, full length cDNA of *GAMYBL2* gene was amplified and cloned into p1301-Nos vector to fuse with Flag through digestion by *BamH*I and *Bcul*I. For GAMYBL2KO plants construction, guider DNA was synthesized and cloned in the pOs-sgRNA vector, and then transferred to the pH-Ubi-cas9-7 vector through LR reaction. Primers and gDNAs used for *GAMYBL2* were listed in Additional file 2: Table S1.

Plasmids were respectively transformed into ZH11 through *Agrobacterium*-mediated genetic transformation in Towin Biotechnology Company (www.towinbio.com/).

### BPH Resistance Detection and Measurements

Individual test assay was carried out at seedling stage using at least six replicates of each cultivar or line as previously described (Wang et al. 2012; Zhao et al. 2016). Each seedling about 5 weeks was infested with twelve second-instar BPH nymphs. Plant status were checked daily, and about 5–12 days later, the plants were scored as susceptible (dead) or resistant (alive). Plant materials were photographed using a Canon EOS7D digital camera.

For small population assay, about 40 plants of tested lines and the WT were planted in a plate in the mud for one month till about third-leaf stage, and fed to BPH population in appropriately 10–15 first-instar nymphs per plant, and the plant status (alive or dead) were surveyed daily in the following days. Plant materials were photographed using a Canon EOS7D digital camera, and the survival rates was calculated based on data from at least three repeats.

Honeydew assay was carried out basically as described (Du et al. 2009).

### RNA Isolation and Quantitative Real-Time RT–PCR (qRT–PCR) Analysis

For gene expression, such as *GAMYBL2*, *OsGAMYB*, and *GS3*, seedlings were used. Total RNAs were extracted using TRIzol (Life technologies, USA) and reverse transcribed using the First Strand cDNA Synthesis Kit (Toyobo). qRT–PCR was performed with the SYBR Green Real-time PCR Master Mix Kit (Toyobo), cDNA was synthesized from 1 μg of total RNA and 1 μl of cDNA was used as template for real-time analysis. The *actin* gene was used as an internal control for normalization. Data from three biological repeats were collected, and the mean value with standard error was plotted.

For gene expression analysis responsive to BPH, around 5-week-old rice seedlings were individually infested with 12 s-instar BPH nymphs that had been starved for 2 h, leaf sheaths and leaves were collected after 0, 2, 4, 8, 12 and 24 h for RNA extraction, the following reverse transcription and qRT–PCR.

All the primer sequences used in vector construction, qRT–PCR and other analysis in this study were listed in Additional file 2: Table S1.

### miRNA Northern Blot Analysis and Stem-Loop qRT–PCR Analysis

miRNA Northern blot was carried out as previously described (Dai et al. 2019). Specifically, the aboveground parts of about 5-week-old rice seedlings before and after BPH feeding were used for RNA extraction, and the OsmiR159 probes were synthesized with 5′-end Biotin. The blots were incubated at 42 °C for 30 min in the Hybridization Buffer (Ambion). And 50–80 pM probes were added in the hybridization buffer to incubate for one night. 5S rRNA was used as control for RNA loading.

Stem-loop qRT–PCR analysis of miRNA was carried out as described (Chen et al. 2022), using miRNA 1st Strand cDNA Synthesis Kit (Vazyme Biotech, China) and miRNA Universal SYBR qPCR Master Mix (Vazyme Biotech, China). The 2^−ΔΔ*C*T^ method was employed to quantify relative gene expression levels. Mean of internal reference gene *U6* were used for normalization.

### Subcellular Localization of OsGAMYBL2 in the Leaves of Nicotiana Benthamiana

Full length cDNA of *OsGAMYBL2* was amplified and cloned into pCAMBIA1300–eGFP vectors to generate OsGAMYBL2–eGFP. The recombinant vectors were transformed into A. tumefaciens GV3101. The recombinant bacteria were cultured and collected through centrifuge, then re-suspended in infection solution (10 mM MES, 10 mM MgCl2 and 200 μM acetosyringone), and infiltrated into *N.* *benthamiana* leaves. 48 h later, fluorescent signals were monitored using a laser confocal scanning microscope (LSM 880, Zeiss).

## Electrophoretic Mobility Shift Assay (EMSA) Assay

For protein expression and purification, the DNA binding domain of GAMYBL2, that is the R2R3 domain, was cloned into the pET44b vector and transformed into E. coli strain BL21 to produce His-tagged fusion protein. The His-R2R3 fusion protein was induced by adding 0.5 mM isopropyl-d-1-thiogalactopyranoside (IPTG) to the culture medium and incubating the cells for 14 h at 20 °C and purified using Ni-NTA (nitrilotriacetic acid) agarose (*GenScript*) according to the manufacturer’s instructions. The GS3P1 DNA probes from *GS3* promoter were synthesized and cy5 labeled. The DNA probes and proteins were co-incubated in the reaction buffer, purified and incubated with the Cy5-labeled probe at 25 °C for 20 min in EMSA buffer (25 mM Hepes (pH 7.5), 40 mM potassium chloride, 3 mM dithiothreitol, 10% glycerol, 0.1 mM EDTA, 0.5 mg/ml bovine serum albumin, 0.5 mg/ml poly-glutamate). After incubation, the reaction mixture was electrophoresed on a 6% native polyacrylamide gel, and then labeled DNA was detected using a Starion FLA-9000 instrument (Fujifilm, Tokyo, Japan).

### Transcriptional Activity Assay

For transcriptional activity assays, the GAL4/UAS system was used. The transcription factors was cloned into pGreenII0800–LUC vector. The reporter was constructed by inserting the upstream activation sequence (UAS) that was bound by the GAL4 protein into pGreenII0800–LUC vector.

### The GAL4/UAS System

The VP16 transcriptional activation domain and full-length CDSs of OsGAMYBL2 fused with the GAL4 DBD were inserted into the pCAMBIA1300-Nos vector as effectors. and The GAL4 DBD was inserted into the pCAMBIA1300-Nos vector as control.

### Dual luciferase (LUC) Assay

For the binding activity assays, the 1500 bp genomic fragment upstream of the *OsGS3* start codon ATG was cloned into the pGreenII 0800-LUC vector as the reporter. The full-length CDSs of OsGAMYBL2 was cloned into pCAMBIA1300Flag-Nos vector as effectors.The p1301Flag-Nos empty vetor was used as negative control.

In the Transcriptional activity assay, The GAL4/UAS system and the Dual luciferase (LUC) assay, all the recombinant construsts were transformed into Agrobacterium tumefaciens strain GV3101(pSoup-P19). Overnight A. tumefaciens cultures were collected by centrifugation and re-suspended in MS medium to OD600 = 1.0, and incubated at RT for 3 h. The reporter and effectors strains were mixed at the ratio of 1:1 and infiltrated into tobacco (Nicotiana benthamiana) leaves and the negative control was infiltrated into the opposite position on the same leaves. Leaves were collected after 3 days (long day/white light) and infiltrated with 150 lg/mL luciferin solution; images were captured using a CCD camera 5 min later and quantification was performed using Dual-Luciferase Reporter Assay System (Promega, Madison, WI). Three biological repeats were measured for each sample.

### Primer Sequences

All the oligo sequences used in this study were listed in Additional file 2: Table S1.

## Supplementary Information

**Additional file 1.** Supplementary figures.

**Additional file 2.** **Table S1.** Oligo names sequences.

## Data Availability

The datasets used and/or analyzed during the current study are available from the corresponding author on reasonable request.
