# Supplementary material for: OsmiR159 Modulate BPH Resistance Through Regulating G-Protein γ Subunit GS3 Gene in Rice
Source: Rice (N Y). 2023 Jul 4;16:30. doi: 10.1186/s12284-023-00646-z (PMC10319700; doi:10.1186/s12284-023-00646-z)

**OsmiR159 modulate BPH resistance through regulating G-protein γ subunit *GS3* gene in rice**

Yanjie Shen^1, 2#^, Guiqiang Yang^3^, Xuexia Miao^1*^, Zhenying Shi^1*^

**Address:**

^1^ Key Laboratory of Insect Developmental and Evolutionary Biology, CAS Center for Excellence in Molecular Plant Sciences, Institute of Plant Physiology and Ecology, Chinese Academy of Sciences, Shanghai, 200032, China

^2^ University of Chinese Academy of Sciences, Shanghai, 200032, China

^3^Wuzhou Agricultural Product Quality and Safety integrated Test Center, Wuzhou, China

**The corresponding author:** zyshi@cemps.ac.cn

**Phone:** +86-21-54924217, Fax: +86-21-54924015

**E-mail:** [zyshi@cemps.ac.cn](mailto:zyshi@cemps.ac.cn)

**Supplementary Figures**

**Figure S1 Detection of STTM159n plants against BPH**

(a) Expression of OsmiR159d in STTM159n and WT plants NIP revealed by stem–loop qRT–PCR (n = 3). Asterisks indicated significant differences compared with NIP plants as determined by Student’s *t*-test (**, *P* < 0.01), with the expression level in ZH11 was set as 1.0. (b) The plant status of STTM159n plants and control NIP plants after BPH infestation for about 7 days in an individual test assay.


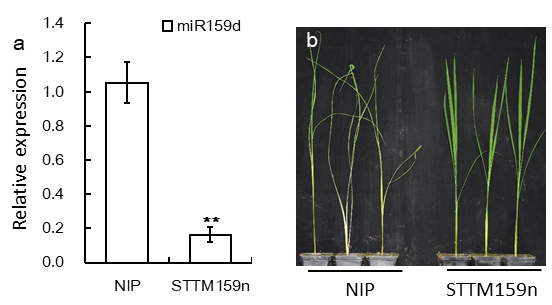


**Figure S2 Expression of *GAMYBL2* gene in the miR159dOE lines and ZH11.**

The expression level in ZH11 was set as 1.0, and asterisks indicate significant differences comparing with that in ZH11 as determined by Student’s *t*-test (**, *P* < 0.01).

**
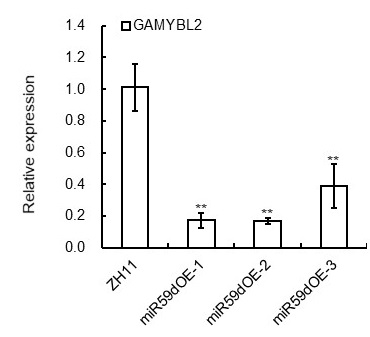
**

**Figure S3 Sketch map of the edited nuclear tides in the *GAMYBL2KO* plants and verification of the GAMYBL2RNAi plants by GAMYBL2 expression.**

(a) Sketch map of the edited nuclear tides in the *GAMYBL2KO* plants. (b) Expression of *GAMYBL2* in the GAMYBL2RNAi and ZH11 lines. The expression level in ZH11 was set as 1.0, and asterisks indicate significant differences comparing with that in ZH11 as determined by Student’s *t*-test (**, *P* < 0.01).


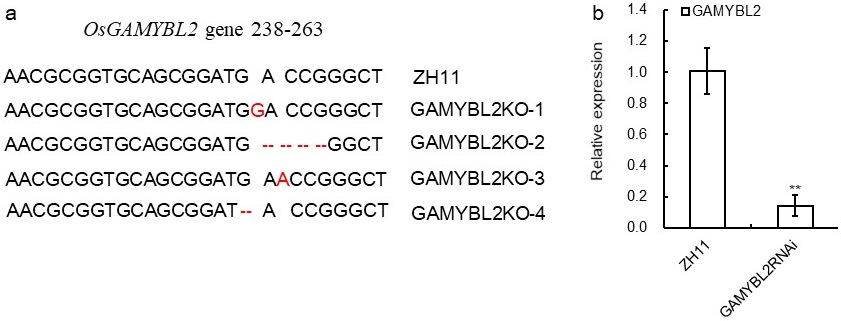


**Figure S4 Sketch map of the structures of the effectors and reporter used in the GAL4/UAS system**


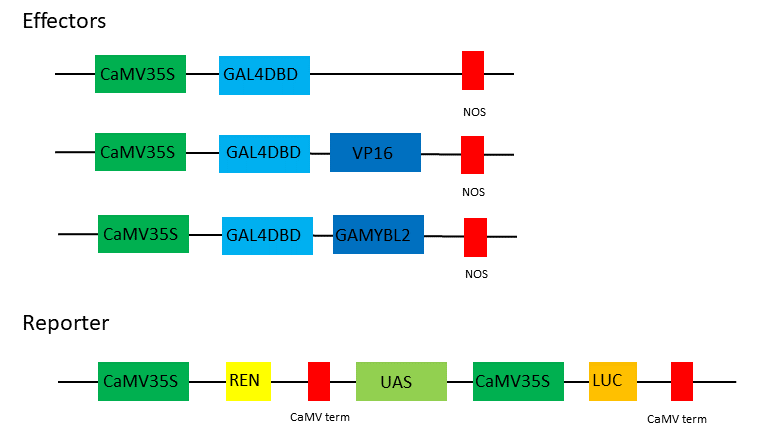


**Figure S5 Determination of the binding motifs of GAMYBL2 protein**

(a) Prediction of the binding motifs of AtMYB101 on the JASPAR website. (b) EMSA of the DNA-binding domain of OsGAMYBL2 binding to the TAACCG motifs as indicated by the blue nulear tides (nts) on the above; (c) EMSA of the DNA-binding domain of OsGAMYBL2 binding to the mutant GGGTCG motifs, the mutant nts were indicated in blue on the above.


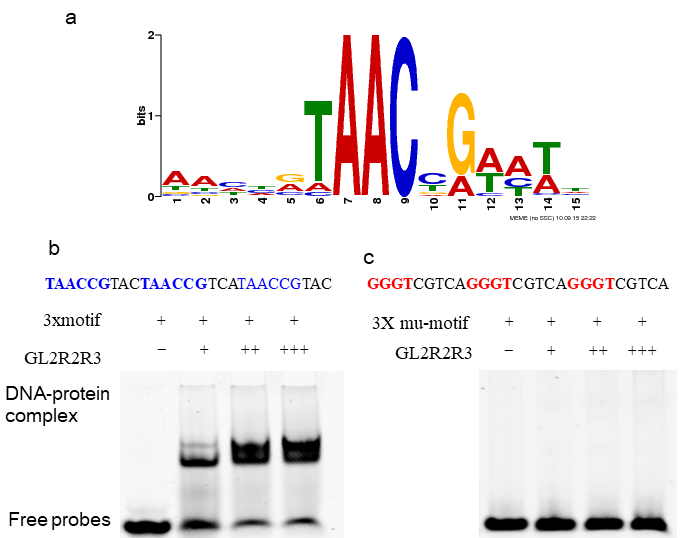


**Figure S6 Sketch map of the 2 Kb promoter of *GS3* gene showing the putative binding motifs of GAMYBL2 protein**


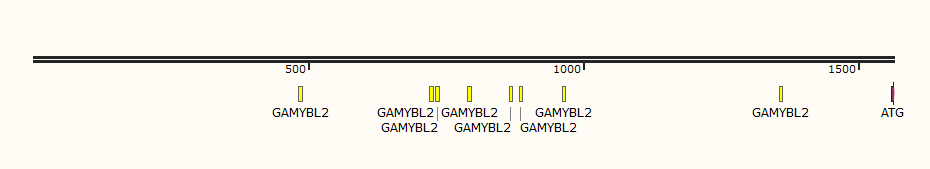


**Figure S7 Verification of the GS3-1OE, GS3-4OE and GS3KO plants**

(a) Expression of *GS3* gene in the GS3-1OE, GS3-4OE and ZH11 plants. The expression level in ZH11 was set as 1.0, and asterisks indicate significant differences comparing with that in ZH11 as determined by Student’s *t*-test (**, *P* < 0.01).

(b) Sequencing of the genomic sequence of the GS3 gene in GS3KO and ZH11 plants. The edited sites (inserting A) were indicated as red.

**
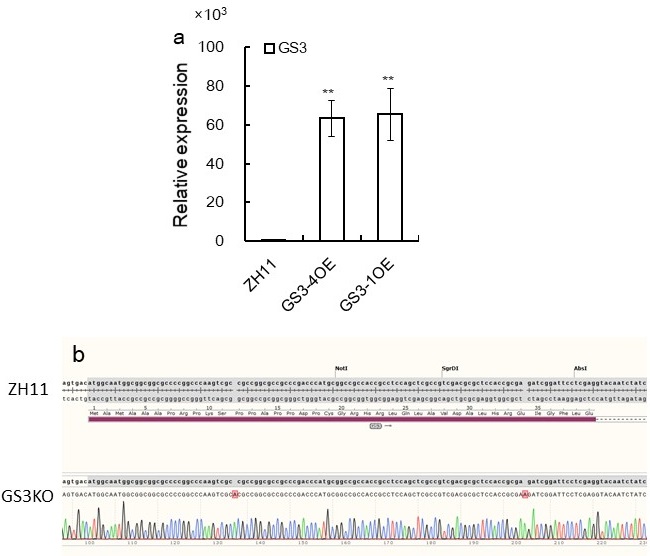
**

**Figure S8 Small population test of the GS3-4OE, ZH11 and GS3KO plants in parallel**

(a) Small population test of the GS3-4OE, ZH11 and GS3KO plants in parallel at about 7 days after BPH infestation.

(b) Small population test of the GS3-4OE, ZH11 and GS3KO plants in parallel at about 10 days after BPH infestation.


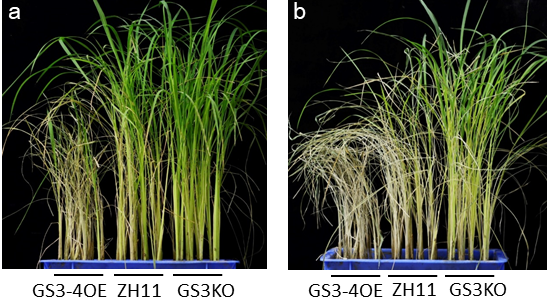


**Figure S9 Expression of *GAMYBL2* and *GS3* gene in STTM159 plants before and after BPH infestation.**

(a) Expression of *GAMYBL2* in STTM159 plants before and after BPH infestation.

(b) Expression of *GS3* in STTM159 plants before and after BPH infestation. The expression level in “0h” was set as 1.0, and asterisks indicate significant differences comparing with that in “0h” as determined by Student’s *t*-test (**, *P* < 0.01; **, *P* < 0.05).


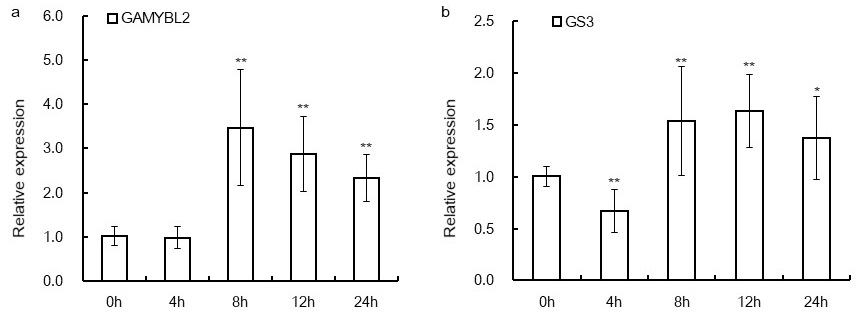

Supplement: Supplementary file 1 — Additional file 1. Supplementary figures. [file 12284_2023_646_MOESM1_ESM.docx]
